# Layer 5 Pyramidal Neurons' Dendritic Remodeling and Increased Microglial Density in Primary Motor Cortex in a Murine Model of Facial Paralysis

**DOI:** 10.1155/2015/482023

**Published:** 2015-05-03

**Authors:** Diana Urrego, Julieta Troncoso, Alejandro Múnera

**Affiliations:** ^1^Behavioral Neurophysiology Laboratory, Universidad Nacional de Colombia, 111321 Bogotá, Colombia; ^2^Department of Molecular Biology of Neuronal Signals, Max-Planck Institute for Experimental Medicine, 37075 Goettingen, Germany; ^3^Department of Biology, School of Sciences, Universidad Nacional de Colombia, 111321 Bogotá, Colombia; ^4^Department of Physiological Sciences, School of Medicine, Universidad Nacional de Colombia, 111321 Bogotá, Colombia

## Abstract

This work was aimed at characterizing structural changes in primary motor cortex layer 5 pyramidal neurons and their relationship with microglial density induced by facial nerve lesion using a murine facial paralysis model. Adult transgenic mice, expressing green fluorescent protein in microglia and yellow fluorescent protein in projecting neurons, were submitted to either unilateral section of the facial nerve or sham surgery. Injured animals were sacrificed either 1 or 3weeks after surgery. Two-photon excitation microscopy was then used for evaluating both layer 5 pyramidal neurons and microglia in vibrissal primary motor cortex (vM1). It was found that facial nerve lesion induced long-lasting changes in the dendritic morphology of vM1 layer 5 pyramidal neurons and in their surrounding microglia. Dendritic arborization of the pyramidal cells underwent overall shrinkage. Apical dendrites suffered transient shortening while basal dendrites displayed sustained shortening. Moreover, dendrites suffered transient spine pruning. Significantly higher microglial cell density was found surrounding vM1 layer 5 pyramidal neurons after facial nerve lesion with morphological bias towards the activated phenotype. These results suggest that facial nerve lesions elicit active dendrite remodeling due to pyramidal neuron and microglia interaction, which could be the pathophysiological underpinning of some neuropathic motor sequelae in humans.

## 1. Introduction

After peripheral nerve axotomy, injured motoneurons undergo both morphological and physiological modifications, including dendritic branches retraction [1, 2], enhanced brain-derived neurotrophic factor [3, 4], c-Fos, and c-Jun expression [5, 6], and changes regarding their active and passive electrophysiological properties [7–9]. Such modifications apparently facilitate motoneuronal survival and the regeneration of their axons. However, depending on whether it is central or peripheral, axotomy would induce motoneuronal death [10]. The mechanisms involved in axotomy induced motoneuronal death are not well understood; however, the loss of muscle fiber-derived trophic factors may be partly responsible [11]. Peripheral nerve lesions have been used for evaluating injury-related neural plasticity not only in motoneurons but also in multiple brain structures. In fact, facial nerve axotomy in rodents has been widely used for studying vibrissal motor system plasticity at different levels [10].

It has been described that a layer 5 pyramidal neuron subpopulation in the vibrissal primary motor cortex (vM1) sends a monosynaptic projection to facial nucleus motoneurons [12]. Although, given such connectivity, it is plausible that facial motoneuron axotomy could transsynaptically induce modifications in vM1, such changes have scarcely been explored. In this vein, it has been described that peripheral facial nerve injury induces astroglial activation in vM1 and other cortical areas as evidenced by transient enhancement of S-100 protein, glial fibrillary acidic protein, and connexin 43 expression [13]. Using c-Fos immunoreactivity as a marker for neuronal activation, it has been found that vM1 volume acutely responding to facial nerve axotomy in sighted rats is twofold smaller than in blind animals and that the degree of acute cortical activation is directly related to the degree of late motor recovery [14]. Significant retraction of layer 5 pyramidal neuron dendritic arborization in vM1 after contralateral facial nerve transection has been described using Golgi-Cox staining [15]. Persistent changes in the electrophysiological properties of layer 5 pyramidal neurons in the primary motor cortex (vM1) induced by facial nerve lesions have been described recently [16]. These changes included increased dendritic excitability of layer 5 pyramidal neurons that could be explained by membrane surface loss due to retraction of their dendritic arborization.

On the other hand, it has been shown that both axotomized [17] and denervated neurons undergo remodeling of their dendritic arborizations [18] following central nervous system (CNS) lesions. Additionally, it has been described that both axotomized and denervated neurons in the CNS display enhanced chemokine expression. Moreover, chemokines activate resident microglial cells and such activation is necessary for dendritic remodeling to occur [18]. It has also been shown that microglial accumulation around the perikarya of axotomized neurons in the CNS is closely related to axonal regeneration [19].

Taking into account the timing of facial nerve lesion induced changes in pyramidal neurons found in previous experiments of our group, the present study was aimed at evaluating the dendritic arborization of layer 5 pyramidal neurons and the density and morphology of the microglia surrounding them in vM1 at one and three weeks after contralateral facial nerve axotomy. To do so, TgH(CX3CR1-EGFP)xTgN(THY1.2-EYFP) transgenic mice were used as they express enhanced yellow fluorescent protein in pyramidal neurons and enhanced green fluorescent protein in microglial cells. Our results showed that peripheral facial nerve axotomy induced persistent shrinking of apical and basal dendrites of layer 5 pyramidal cells in vM1, accompanied by a significant increase of neighboring microglia density with a shift towards activated phenotype.

## 2. Experimental Procedures

### 2.1. Subjects

Twelve adult male TgH(CX3CR1-EGFP)xTgN(THY1.2-EYFP) mice, weighing 23 ± 1.5 g (mean ± standard error of the mean (SEM)), kindly supplied by the Glial Physiology and Imaging Group (Department of Neurogenetics, Max Planck Institute for Experimental Medicine, Göttingen, Germany) were used as subjects. The animals were housed in the Max Planck Institute for Experimental Medicine (Göttingen, Germany) mouse facility, having* ad libitum* access to food and water, and being kept in a sound-attenuated room with controlled humidity (40 ± 5%) and temperature (20 ± 1°C) using a 12 h light/dark cycle (lights on at 07:00 a.m.). All experimental procedures were performed according to the Max-Planck-Society and European guidelines for the welfare of experimental animals and were approved by the local Ethics Committee. All efforts were made to minimize the number of animals used and avoid unnecessary suffering to the experimental subjects.

TgH(CX3CR1-EGFP)xTgN(THY1.2-EYFP) mice were obtained by crossbreeding homozygous CX3CR1-EGFP mice, in which enhanced green fluorescent protein (EGFP) expression in microglia is achieved by placing the EGFP reporter gene into the Cx3cr1 locus encoding the chemokine receptor CX3CR1 [20], with transgenic THY1.2-EYFP mice expressing enhanced yellow fluorescent protein (EYFP) in projection neurons and their respective axons [21]. TgH(CX3CR1-EGFP) mice and TgN(THY1.2-EYFP) mice were of B6SJL background for more than 10 generations.

### 2.2. Facial Nerve Lesion Surgery

**Figure 1:**
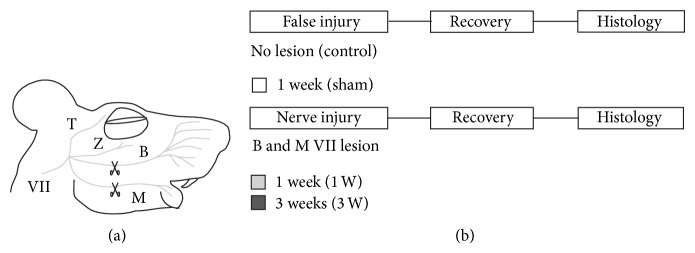
Experimental preparation and design. (a) Lateral view of a mouse's head, schematizing facial nerve branches innervating face muscles; the branches transected during irreversible nerve lesion surgery, as well as the approximate place where they were cut, are indicated by scissors. T, temporal; Z, zygomatic; B, buccal; and M, mandibular branches of VII facial nerve. (b) Schema of the experimental design showing its time line and indicating the color representing each group (white for sham, light gray for 1 week, and black for 3 weeks after facial nerve lesion).

Eight randomly chosen mice underwent facial nerve transection surgery in aseptic conditions and under general anesthesia (100 mg/kg ketamine and 10 mg/kg xylazine, administered intraperitoneally). Briefly, the facial nerve's buccal branch and the upper division of the marginal mandibular branch were dissected and severed through a 0.5 cm horizontal incision made above the right mandibular angle; a 2 mm segment of the proximal stump was removed in each sectioned nerve branch to avoid nerve repair and the skin wound was sutured with discontinuous 5-0 silk stitches (Figure 1(a)). Nerve branch identity and lesion effectiveness were confirmed by electrical stimulation [22].

Four randomly chosen subjects underwent sham surgery (control). Briefly, the buccal branch and the upper division of the marginal mandibular branch of the facial nerve were dissected through a 0.5 cm horizontal incision above the right mandibular angle, but left intact; the skin wound was sutured with discontinuous 5-0 silk stitches.

### 2.3. Histology

Subjects undergoing facial nerve axotomy were allowed a recovery period of either 1 week (*n* = 4) or 3 weeks (*n* = 4) before being sacrificed for histological analysis. Sham-operated subjects (*n* = 4) were allowed a one week recovery period before being killed for histological analysis (Figure 1(b)). After the recovery time had elapsed, the mice were deeply anesthetized with isofluorane (1.5–2.5%; mixed with 0.6 to 0.8 liters/min O_2_) and transcardially perfused with Hank's balanced salt solution (HBSS, Gibco) followed by 4% paraformaldehyde in 0.1 M phosphate buffered saline. The brains were dissected after the perfusion and postfixed overnight in 4% paraformaldehyde in 0.1 M phosphate buffered saline at 4°C. Serial 80 *μ*m thick coronal slices (1 to 2.5 mm rostral to bregma) containing the vM1 (i.e., medial agranular cortex, from midline to 1.5 mm lateral) were obtained using a vibratome (Leica VT 1000S, Leica Instruments, Germany).

### 2.4. Microscopy

Image stacks of the fixed brain sections were recorded using a two-photon laser scanhead (TriM-Scope, La Vision Biotec, Bielefeld, Germany) coupled to a fixed stage upright microscope (Axioscope 2 FS, Zeiss, Oberkochen, Germany). Two-photon excitation at 910 nm achieved by a titanium-sapphire laser equipped with broadband optics (MaiTai BB, Spectra Physics) and emission fluorescence detection through a 530–600 nm band pass filter were used for detecting pyramidal neurons expressing EYFP. Excitation at 970 nm and fluorescence detection through a 500–530 nm band pass filter were used for detecting microglial cells expressing EGFP. Non-descanned detection (Hamamatsu, Japan) was used to record three-dimensional image stacks having 1,024 pixel × 1,024 pixel frame size (200 *μ*m × 200 *μ*m scan field) through a 40x, 1.3 numerical aperture oil objective (Zeiss).

### 2.5. Data Processing and Analysis

EYFP-expressing pyramidal neurons from layer 5 in vM1 were identified by their characteristic triangular soma shape, apical dendrites oriented towards the pial surface, and presence of dendritic spines. Image stacks containing the dendritic arborization of a given pyramidal neuron were then overlapped using ImageJ (http://rsb.info.nih.gov/ij/). Three layer 5 vM1 neurons whose dendritic trees were extensively represented in the overlapped stack were then selected for every subject for reconstruction using Neuromantic 1.6.3, an open source system for three-dimensional digital tracing of neurites [23].

The three-dimensionally reconstructed dendritic arborizations were then compressed to a single plane and skeletonized using ImageJ routines for Sholl analysis [24]. Briefly, concentric circles were traced, starting from the soma, increasing their radius by 4 *μ*m, and then the branches intersected by each circle were counted using an ImageJ routine. A plot illustrating how the number of branches varied as a function of distance from the soma was constructed and the area under the curve (AUC), the maximum, and the full width half maximum (FWHM) were calculated [25].

Dendritic spine density was estimated by counting the number of spines found in a randomly selected 10 *μ*m long segment of a dendritic branch. Dendritic branches were classified as either apical or basal and then as primary, secondary, or tertiary. An ImageJ routine was used for spine counting.

Microglial cells from layer 5 in vM1 were identified by their EGFP expression. Image stacks containing only layer 5 of vM1 (to do so, a region of interest encompassing 500 to 1000 *μ*m from pial surface in the medial agranular cortex was defined) were selected for analysis. Microglial cells per volume unit density and the area of each cell's soma were measured using automated ImageJ analyse particles routine (16-bit grayscale images' contrast was enhanced to discriminate individual cells, and then size and shape criteria were set to exclude noisy particles).

Differences among groups were statistically evaluated for every parameter using SigmaPlot 12.0 (Systat Software Inc., Chicago, USA) one-way analysis of variance (ANOVA) module; significance level was set at *P* < 0.05. Whenever ANOVA revealed a significant difference, Holm-Sidak* post hoc* multiple pairwise *t*-tests were performed to identify the source of such difference.

## 3. Results

### 3.1. Vibrissal Paralysis

Unilateral section of the buccal and mandibular facial nerve branches induced complete paralysis of the right mystacial vibrissae, which persisted for at least three weeks. The whiskers were oriented backwards during the first recovery week, forming a narrow bunch; from that time on, the whisker bunch progressively loosened but still remained immobile. Sham-operated mice whisking was not distinguishable from that of nonoperated mice (data not shown).

### 3.2. Facial Nerve Lesion Induced Dendritic Remodeling of vM1 Pyramidal Neurons

**Figure 2:**
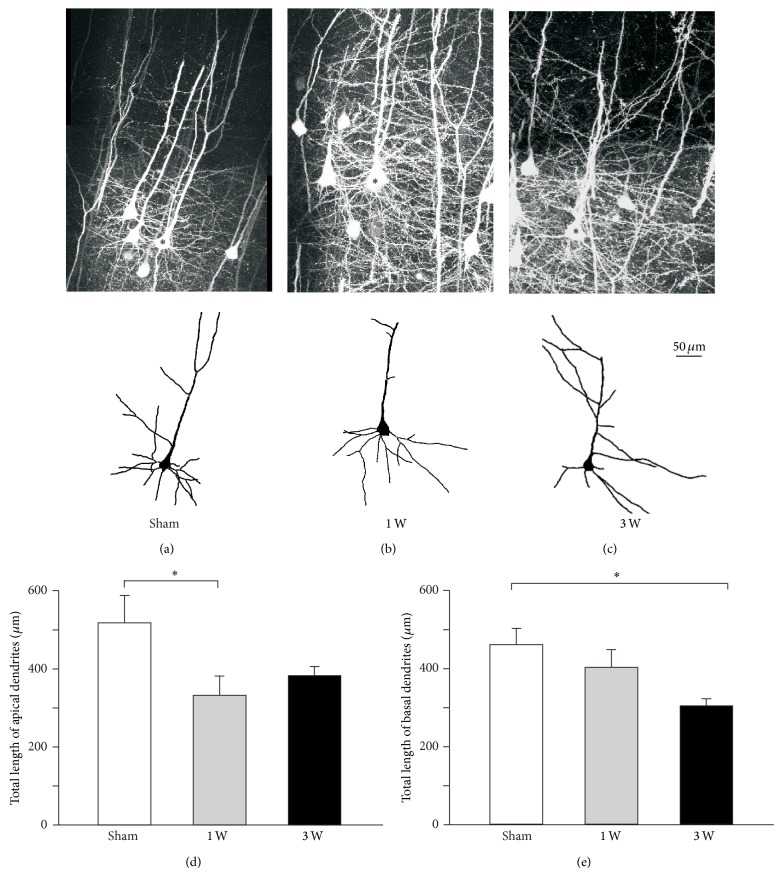
Differential apical and basal dendrite length modifications in vM1 layer 5 pyramidal neurons following facial nerve axotomy. (a, b, c) Top row, two-dimensional projection of stacks of two-photon confocal images of vibrissal motor cortex layer 5 pyramidal neurons expressing EYFP; bottom row, two-dimensional computer-assisted traces of layer 5 pyramidal neurons from sham and injured animals (reconstructed neurons are indicated by an asterisk in top row images). Micrographs and reconstructions obtained either from mice undergoing (a) sham surgery, (b) one week recovery after facial lesion, and (c) three-week recovery after facial lesion. (d) Total length of apical dendritic tree of layer 5 pyramidal neurons from each experimental group. (e) Total length of basal dendritic tree of layer 5 pyramidal neurons from each experimental group. Bars and error whiskers represent the mean + SEM. 1 W, 1 week after peripheral nerve lesion; 3 W, 3 weeks after peripheral nerve lesion; ^∗^ *P* < 0.05.

Facial nerve axotomy induced changes in the dendritic architecture of pyramidal neurons of the layer 5 in contralateral vM1 (Figure 2). The total length of the apical dendrites changed significantly after facial nerve injury (*F*_(2,   33)_ = 3.500, *P* = 0.042). One week after the injury (Figure 2(d)) apical dendrites became pruned and their total length became significantly shorter than that of sham-operated subjects (*t* = 2.560, *P* = 0.045). Apical dendrites had partially regrown three weeks after the lesion (Figure 2(d)) and their total length was not significantly different from that of control subjects (*t* = 1.858, *P* = 0.139).

Facial nerve axotomy also induced significant changes in the total length of basal dendrites of pyramidal neurons in contralateral vM1 (*F*_(2,  33)_ = 4.475, *P* = 0.019). One week after the lesion (Figure 2(e)) basal dendrites' total length was not significantly different from that observed in sham-operated subjects (*t* = 1.093, *P* = 0.283). However, three weeks after the injury (Figure 2(e)), the basal dendrites became pruned and their total length became significantly shorter than that of control subjects (*t* = 2.958, *P* = 0.017).

Although the apical to basal total length ratio did not significantly change after facial nerve injury (*F*_(2,  33)_ = 2.161, *P* = 0.131), the correlation between apical and basal dendrites' total length disappeared after facial nerve axotomy. In fact, control subjects' total apical dendrite length was positively and significantly correlated to total basal dendrite length (*r* = 0.970, *P* = 0.001). Due to the changes described above regarding apical and basal dendritic branches, such correlation became weaker and nonsignificant both one week (*r* = 0.312, *P* = 0.324) and three weeks (*r* = −0.231, *P* = 0.659) after facial nerve section.

**Figure 3:**
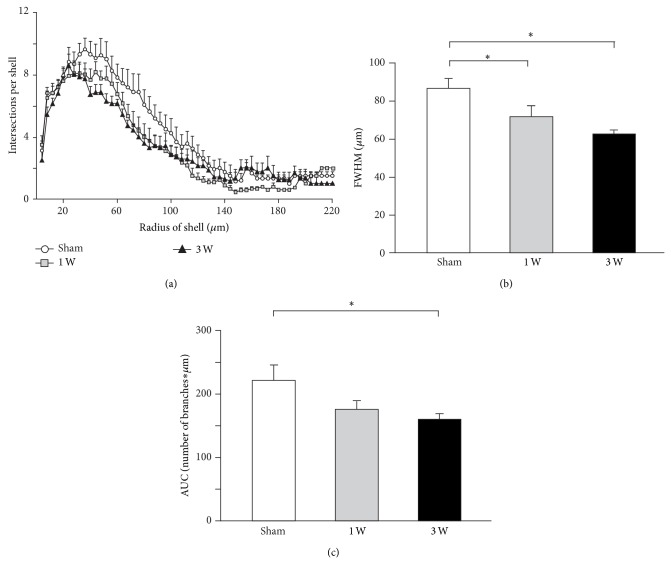
Overall dendritic tree shrinkage in vM1 layer 5 pyramidal neurons following contralateral facial nerve axotomy. (a) Sholl analysis indicating the number of dendrite branches crossing concentric circles around the cell body. Symbols and associated error bars correspond to the mean ± SEM. (b) Full width half maximum (FWHM) of Sholl distribution from each experimental group. Bars and error whiskers represent the mean + SEM. (c) Sholl distribution area under the curve (AUC) from each experimental group. Bars and error whiskers represent the mean + SEM. 1 W, 1 week after peripheral nerve lesion; 3 W, 3 weeks after peripheral nerve lesion; ^∗^ *P* < 0.05.

The dendritic arborization of vM1 layer 5 pyramidal neurons shrank globally after contralateral facial nerve axotomy, as indicated by Sholl analysis of the distribution of the number of dendritic branches as a function of distance from the soma (Figure 3). The maximum for such distribution did not significantly change after facial nerve lesion (Figure 3(a); *F*_(2,  33)_ = 1.032, *P* = 0.368). However, the FWHM became significantly smaller than control from the first week after facial nerve axotomy onwards and remained so for at least three weeks (Figure 3(b); *F*_(2,  33)_ = 7.468, *P* = 0.002; sham versus 1 week: *t* = 2.369, *P* = 0.047; sham versus 3 weeks: *t* = 3.829, *P* = 0.002). Meanwhile, the area under the curve for such distribution significantly changed after facial nerve section (Figure 3(c); *F*_(2,  33)_ = 3.844, *P* = 0.032), becoming significantly smaller than control after three weeks (sham versus 1 week: *t* = 1.986, *P* = 0.108; sham versus 3 weeks: *t* = 2.669, *P* = 0.035).

### 3.3. Facial Nerve Axotomy Induced Dendritic Spine Pruning in vM1 Pyramidal Neurons

**Figure 4:**
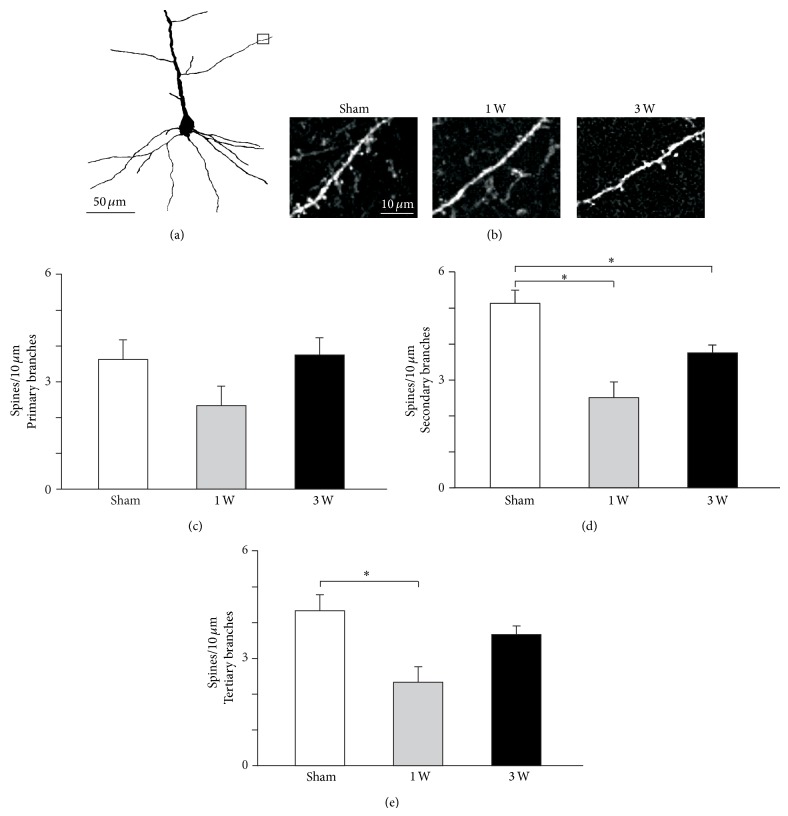
Apical dendritic spine density changes in vM1 layer 5 pyramidal neurons following facial nerve axotomy. (a) Two-dimensional computer-assisted trace of layer 5 pyramidal neuron from a representative mouse sacrificed 1 week after facial nerve lesion. The small rectangle indicates the area photographed in (b). (b) Representative microphotographs of second order dendritic spines from each experimental group. (c, d, e) Quantification of layer 5 pyramidal neurons spine density in 1st, 2nd, and 3rd order apical dendrites for each experimental group. Bars and error whiskers represent the mean + SEM. 1 W, 1 week after peripheral nerve lesion; 3 W, 3 weeks after peripheral nerve lesion; ^∗^ *P* < 0.05.

The density of dendritic spines of vM1 layer 5 pyramidal neurons became significantly reduced after contralateral facial nerve injury (Figure 4). Spine pruning occurred in secondary and tertiary apical dendrite branches, but not in their primary branches (Figure 4(c); *F*_(2, 33)_ = 2.280, *P* = 0.118). Spine density in secondary apical dendrite branches became significantly reduced after facial nerve lesion and remained so for at least three weeks (Figure 4(d); *F*_(2, 33)_ = 14.345, *P* < 0.001; sham versus 1 week: *t* = 5.354, *P* < 0.001; sham versus 3 weeks: *t* = 2.805, *P* = 0.017). By contrast, dendritic spine pruning in the tertiary apical dendrite branches occurred only one week after facial nerve lesion but not three weeks later (Figure 4(e); *F*_(2, 33)_ = 7.000, *P* = 0.003; sham versus 1 week: *t* = 3.674, *P* = 0.003; sham versus 3 weeks: *t* = 1.225, *P* = 0.229).

**Figure 5:**
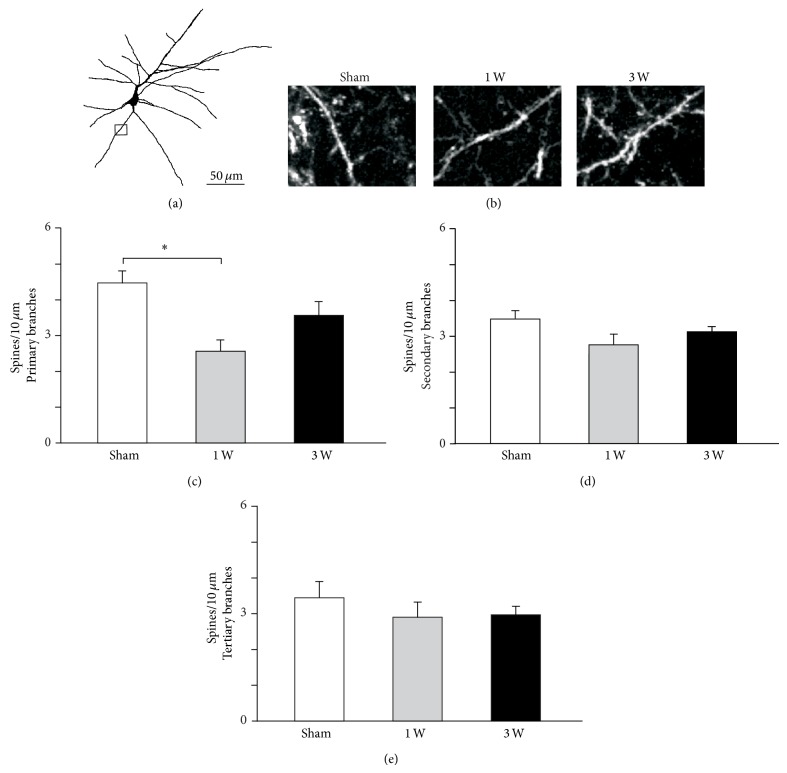
Basal dendritic spine density changes in vM1 layer 5 pyramidal neurons following facial nerve axotomy. (a) Two-dimensional computer-assisted trace of layer 5 pyramidal neuron from a representative mouse sacrificed 1 week after facial nerve lesion. The small rectangle indicates the area photographed in (b). (b) Representative microphotographs of first order dendritic spines from each experimental group. (c, d, e) Quantification of layer 5 pyramidal neurons spine density in 1st, 2nd, and 3rd order basal dendrites for each experimental group. Bars and error whiskers represent the mean + SEM. 1 W, 1 week after peripheral nerve lesion; 3 W, 3 weeks after peripheral nerve lesion; ^∗^ *P* < 0.05.

Contralateral facial nerve injury-induced spine pruning in the basal dendrites of vM1 layer 5 pyramidal neurons (Figure 5) was restricted to primary branches (Figure 5(c); *F*_(2, 33)_ = 8.422, *P* = 0.001) and occurred transiently during the first week after the lesion (sham versus 1 week: *t* = 4.099, *P* < 0.001; sham versus 3 weeks: *t* = 1.871, *P* = 0.070). There was a nonsignificant tendency in secondary basal dendrite branches towards decreased density of dendritic spines after contralateral facial nerve section (Figure 5(d); *F*_(2, 33)_ = 3.256, *P* = 0.051). By contrast, spine density in tertiary basal dendrite branches did not significantly change after the injury (Figure 5(e); *F*_(2, 33)_ = 2.035, *P* = 0.217).

### 3.4. Facial Nerve Section Induced Increased Microglial Density around vM1 Pyramidal Neurons

**Figure 6:**
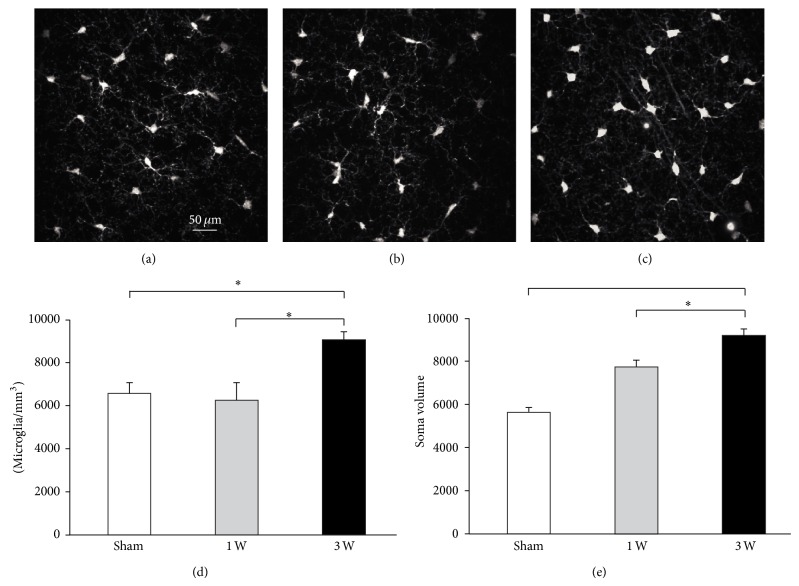
Increased microglial cell density and soma area surrounding vM1 layer 5 pyramidal neurons following facial nerve axotomy. (a, b, c) Microphotographs of microglia cells around vM1 layer 5 pyramidal neurons from representative sham (a), 1 week (b), and 3 weeks (c) mice. Quantification of cell density (d) and soma area (e) for microglial cells surrounding vM1 layer 5 pyramidal neurons from each experimental group. Bars and error whiskers represent the mean + SEM. 1 W, 1 week after peripheral nerve lesion; 3 W, 3 weeks after peripheral nerve lesion; ^∗^ *P* < 0.05.

Evidence was found of microglial recruitment and bias towards activated phenotype surrounding vM1 layer 5 pyramidal neurons (Figure 6). The density of microglial cells surrounding vM1 layer 5 pyramidal neurons changed significantly after contralateral facial nerve lesion (Figure 6(d); *F*_(2, 33)_ = 18.394, *P* < 0.001). One week (Figure 6(b)) after the axotomy there were no significant changes in microglial cell density (Figure 6(d); sham versus 1 week: *t* = 0.625, *P* = 0.536) with respect to that found in control subjects (Figure 6(a)). By contrast, three weeks after facial nerve injury (Figure 6(c)), heightened density was found for microglial cells surrounding vM1 layer 5 pyramidal neurons (Figure 6(d); *t* = 4.912, *P* < 0.001).

The morphology of the microglial cells surrounding vM1 layer 5 pyramidal neurons changed significantly after contralateral facial nerve axotomy (Figure 6(e); *F*_(2, 33)_ = 5.161, *P* = 0.011). One week after the lesion (Figure 6(b)) there was a nonsignificant increase in the soma of microglial cells with respect to that found in control subjects (Figure 6(e); *t* = 1.890, *P* = 0.131). By contrast, three weeks after the axotomy (Figure 6(c)), microglial cell somata became significantly larger than control (Figure 6(e); *t* = 3.195, *P* = 0.009). Also, three weeks after the injury, the processes of microglial cells became shorter and became oriented surrounding layer 5 pyramidal cells dendrites (Figure 6(c), arrows).

## 4. Discussion

This research has shown that an irreversible lesion of the buccal and mandibular branches of the facial nerve induced not only complete and permanent vibrissal paralysis, but also progressive and long-lasting changes in the dendritic morphology of vM1 layer 5 pyramidal neurons as well as increased density and a shift towards activated phenotype in the surrounding microglia.

Our group has previously used the Golgi-Cox staining technique to show that irreversible peripheral facial nerve lesion in rats induces a significant retraction of the dendritic arborization of layer 5 pyramidal neurons in contralateral vM1 [15]. The present work has further characterized the dynamics of dendritic arborization remodeling, using two-photon confocal microscopy to scan EYFP-expressing pyramidal neurons of layer 5 in the vM1 of mice submitted to irreversible facial nerve lesion. The dendritic arborization of the pyramidal cells underwent overall shrinkage, persisting for at least three weeks; however, such shrinkage was not homogeneous in space or time. In fact, apical dendrites underwent transient shortening one week after facial nerve lesion, which became almost completely reverted by the third week. By contrast, basal dendritic remodeling followed an inverse pattern after facial nerve section; although basal tree became progressively shorter, such change only became significant three weeks after axotomy. In addition, this work also led to finding that vM1 layer 5 pyramidal neurons underwent dendritic spine pruning after facial nerve injury occurring in distal apical and in proximal basal branches. vM1 layer 5 pyramidal neurons receive segregated sensory and motor input; somatosensory input, coming from primary somatosensory cortex and posteromedial thalamic nucleus, is primarily directed towards distal apical dendrite branches, while motor input, coming from M2, orbital cortex and motor thalamic nuclei, is primarily directed towards proximal basal dendrite branches and soma [26]. Horizontal projections from neighboring cortical columns are mainly distributed in layer 2/3, therefore impinging on apical dendrites of layer 5 pyramidal neurons [27]. Layer 5 pyramidal neurons' basal dendrites also receive recurrent excitatory input from axons of projecting neurons [28] and it has been suggested that such recurrent input synchronizes the firing of multiple corticofacial neurons during the generation of vibrissal motor commands [29]. Transient branch retraction and spine pruning in distal apical dendrites may therefore have been related to an initial retraction of regular somatosensory input succeeded by invasion by either horizontal projections from neighboring cortical columns or a different set of somatosensory input, which may have shifted commitment regarding somatosensory information. In addition, progressive retraction and spine pruning in proximal basal dendrites may be related to ongoing disengagement from motor input and synchronizing retrograde projections, which may render vM1 less efficient to generate motor commands.

A previous paper by our group described that vM1 layer 5 pyramidal neurons displayed increased dendritic excitability and decreased response to whisker-pad stimulation after contralateral facial nerve axotomy [16]. Both findings could be related to the structural modifications described herein; while a loss of dendritic membrane would cause increased excitability due to increased input resistance, a loss of synaptic input (as evidenced by dendritic spine pruning) would explain the decreased response to somatosensory stimulation. The aforementioned structural and functional modifications in vM1 layer 5 pyramidal neurons induced by contralateral facial nerve axotomy could have been caused by a loss of synaptic communication with target facial motoneurons and an imbalance in somatosensory information processing induced by vibrissal immobility.

On the one hand, facial motoneuron axotomy, apart from direct cellular damage, interrupts the trophic relationship between motoneurons and the muscle fiber they have used to innervate which, in turn, causes structural, metabolic, electrophysiological, and molecular alterations associated with a survival and regeneration programme, not only in axotomized motoneurons, but also in their surrounding glial cells [10]. As a consequence of such response, vM1 layer 5 pyramidal neurons lose their synaptic targets within the facial nucleus due to progressive dendritic retraction and synaptic stripping of axotomized facial motoneurons, which implies that corticofacial neurons become deprived of the neurotrophic factors released by motoneurons. In fact, pyramidal tract lesion (which interrupts the trophic interaction between corticospinal neurons in the primary motor cortex and their targets) induces a reduction in somatic volume, increased excitability, and reduced inhibitory synaptic input in primary motor cortex layer 5 pyramidal neurons [30]. Moreover, it has been described that the premotor neuron phenotype changes after injury to the motoneurons over which they project [31].

On the other hand, vM1 reorganization after facial nerve lesion depends critically on the alteration of vibrissal somatosensory input. A section of the infraorbitary nerve induces vibrissal representation shrinkage in neonatal rats [32] and a significant increase in the minimum intracortical vM1 stimulation intensity required to evoke vibrissal movements in adult rats [33]. Transiently restricting sensory and motor vibrissal functions, either by total whisker clipping [34] or botulinum toxin application in the whisker pad [35, 36], causes reversible shrinkage of the cortical representation of vibrissal musculature and its invasion by neighboring representations. Taken together, these antecedents suggest that vibrissal paralysis causes a remarkable imbalance in somatosensory input to vM1. Perturbation of whisker paralysis-induced vibrissal sensory function would then cause disruption of somatosensory afferents.

Irreversible facial nerve lesion induced increased density and a shift towards activated phenotype in the microglia surrounding vM1 layer 5 pyramidal neurons; such microglial changes occurred after a delay lasting longer than a week. To our knowledge, this is the first report ever of microglial recruitment and bias towards activated phenotype in vM1 following facial nerve section. The long delay of microglial response described here in vM1 is also noteworthy. In fact, microglial cells become activated by diverse injuries to CNS neurons; such activation occurs within tens of minutes after direct injury, as observed in the cortex after medial cerebral artery occlusion [37]. Microglial activation can follow a slower pace, within days, when lesion occurs far from the neuronal soma, as observed in facial motoneurons following facial nerve axotomy [38]. Microglial activation starts even later in structures secondarily affected by injury, like the thalamus after medial cerebral artery occlusion [37, 39].

It is plausible that the microglial response in vM1 reported here was due to biochemical changes in layer 5 corticofacial neurons induced by retrograde transsynaptic signaling from the axotomized facial motoneurons over which they project. Such retrograde signaling occurs after axotomy, since motor cortex astrocyte activation after peripheral facial nerve lesion has been reported to occur as soon as 1 h after lesion and disappear 5-6 days later [13]. vM1 layer 5 pyramidal cells may thus have induced and controlled microglial activation through the secretion of diverse cytokines. In fact, neuronal secretion of C-X-C motif chemokine 10 elicits microglial activation through interaction with microglial C-X-C chemokine receptor 3 and such activation is essential for dendritic remodeling after axotomy of corticospinal neurons [18]. Additionally, neuronal secretion of fractalkine prevents microglial neurotoxicity through interaction with microglial [40] and neuronal CX3CR1 receptor [41]. Moreover, it has been reported that marked microglial activation occurs around the cell bodies of intrinsic CNS neurons regenerating axons into a peripheral nerve graft, and such activation is closely correlated with axonal regeneration [39]. The observed microglial response in vM1 after facial nerve axotomy might therefore have been related to dendritic remodeling and spine pruning and axon growth towards a new synaptic target.

The facial nerve lesion induced structural changes in vM1 layer 5 pyramidal cells described here imply active reorganization of intrinsic cortical circuitry associated with controlled microglial response. Such reorganization seems to have been directly related to functional changes in vM1 layer 5 pyramidal neurons following facial axotomy, as described elsewhere [16]. Such structural and functional modifications may occur in people with facial palsy and may represent the pathophysiological underpinning of some of the functional sequelae found in them and in patients suffering other peripheral neuropathies. The facial nerve axotomy model is therefore helpful in understanding cortical plasticity related to peripheral nerve injury and the pathophysiology of neuropathic sequelae in human beings.

## 5. Conclusions

The facial nerve lesion induced facial paralysis and structural changes in vM1 layer 5 pyramidal cells that project to facial muscles. These central nervous system changes were long lasting and imply dendritic reorganization of pyramidal neurons and dendritic spine pruning of layer 5 pyramidal neurons. These changes were associated with surrounding microglial response and seem to have been directly related with functional changes in vM1 layer 5 pyramidal neurons. Such structural and functional modifications may occur in people with facial palsy and may represent the pathophysiological underpinning of some of the functional sequelae found in these patients.

## Abbreviations

1 W: 1 week
3 W: 3 weeks
ANOVA: Analysis of variance
AUC: Area under the curve
CNS: Central nervous system
CX3CR1: CX3 chemokine receptor 1
C-X-C: Protein motif containing two cysteine residues separated by any amino acid
EGFP: Enhanced green fluorescent protein
EYFP: Enhanced yellow fluorescent protein
FWHM: Full width half maximum
SEM: Standard error of the mean
THY: Thymocyte differentiation antigen
vM1: Vibrissal primary motor cortex.
